# Supplementary material for: Learning curve and surgical outcome of robotic assisted colorectal surgery with ERAS program
Source: Sci Rep. 2022 Nov 29;12:20566. doi: 10.1038/s41598-022-24665-w (PMC9709162; doi:10.1038/s41598-022-24665-w)
Supplement: Supplementary file 1 — Supplementary Information 1. [file 41598_2022_24665_MOESM1_ESM.zip › LSC Raw data-Ñ[▒K/097/H_ñw╝╨░O▒Kñσ.pdf]

正式摘要(電子簽章：000000201497334)		
W52 / W52 ☐ 英文標題(注意:無英文姓名-請補病人基本檔!)	☐【讀取】憑證	

臺中榮民總醫院
出院病歷摘要

轉入號： 轉出號：

[redacted]

(8)流水編號	
(9)入院日期	109/10/13 (2020) 直腸外科 W52 - 155
(10)轉科日期	
(11)出院日期	109/10/22 (2020) 住院天數計 9 日

診斷

- 入院：1. Squamous cell carcinoma in situ arising in condyloma acuminatum
  s/p total anoplasty on 2015/01/30
  s/p repeated resection and CCRT under our CRS and C/T service
  tumor recurrence s/p resection on 2020/05/28
  , s/p salvage IGRT 25 Gy / 5 fr. to rectal tumor 2020-08-13
  , with tumor recurrence
  2. CIS of cervix, extensive moderate dysplasia and condyloma of bilateral labial major, minor, and perianus
  , s/p conization of the uterine and ECC, skinny vulvectomy on 2009/07/28
  3. SLE diagnosed at age 13, currently under our IMRH OPD for medication
  4. APS diagnosed on 1997/9
  5. Cataract, OU. s/p phaco-IOL + SCI decadron OD on 2020.01.07 ; s/p ECCE + ant VT OS on 1090206, s/p ant VT + 3P in sulcus on 2020.2.20
- 出院：1. Squamous cell carcinoma in situ arising in condyloma acuminatum
  s/p total anoplasty on 2015/01/30
  s/p repeated resection and CCRT under our CRS and C/T service
  tumor recurrence s/p resection on 2020/05/28
  , s/p salvage IGRT 25 Gy / 5 fr. to rectal tumor 2020-08-13
  , with tumor recurrence, s/p laparoscopic APR on 20201015
  2. CIS of cervix, extensive moderate dysplasia and condyloma of bilateral labial major, minor, and perianus
  , s/p conization of the uterine and ECC, skinny vulvectomy on 2009/07/28
  3. SLE diagnosed at age 13, currently under our IMRH OPD for medication
  4. APS diagnosed on 1997/9
  5. Cataract, OU. s/p phaco-IOL + SCI decadron OD on 2020.01.07 ; s/p ECCE + ant VT OS on 1090206, s/p ant VT + 3P in sulcus on 2020.2.20

主訴

- Anal pain with bleeding

病史

- This 47 Y/O female has main hx of :
  1. Squamous cell carcinoma in situ arising in condyloma acuminatum
  s/p total anoplasty on 2015/01/30
  s/p repeated resection and CCRT under our CRS and C/T service
  tumor recurrence s/p resection on 2020/05/28
  , s/p salvage IGRT 25 Gy / 5 fr. to rectal tumor 2020-08-13
  , with tumor recurrence
  2. CIS of cervix, extensive moderate dysplasia and condyloma of bilateral labial major, minor, and perianus
  , s/p conization of the uterine and ECC, skinny vulvectomy on 2009/07/28
  3. SLE diagnosed at age 13, currently under our IMRH OPD for medication
  4. APS diagnosed on 1997/9
  5. Cataract, OU. s/p phaco-IOL + SCI decadron OD on 2020.01.07 ; s/p ECCE + ant VT OS on 1090206, s/p ant VT + 3P in sulcus on 2020.2.20
  Last timr excision on 2020/05/28 showed margin positive. Anal pain with intermittent bleeding was still noted and bothered her life. After discuss with the patient, she was admitted for APR.

體檢發現

-
  - General appearance
    - A well-nourished and well-developed person, with acute ill looking and clear consciousness.

-
  - Vital signs:

date	體溫(BT)	脈搏(P)	呼吸(R)	血壓(BP)	身高(BH)	體重(BW)	BMI
20201013	36.7C	99/min	18/min	101/61mmHg	154.2cm	58.9kg	24.8

  - Body weight and Height: Body weight: not available , Height: not available , BMI: not calculated , Waist circumference: not available
- Skin
  - Normal skin turgor , No jaundice , No change of moisture or temperature , No cyanosis , No rash , No ulcer , No petechiae , No purpura
- Lymphadenopathy
  - No lymphadenopathy
- HEENT
  - Head:
    - No shape deformity , No laceration , No scar , No hair change , No facial change , Normal lip
  - Eyes:
    *Conjunctiva: Normal *Sclera: Not icteric *Cornea: Clear
    *Light reflex: Normal *Movement: Normal *Eyelids: Normal
  - Ears:
    - No deformity , No hearing loss
  - Nose:
    - No discharge from nose , No mucosa change
  - Mouth and throat:
    - No oral mucosa change , No throat injection , No tonsil enlargement
- Neck
  - No stiffness/rigid , No carotid bruit , Jugular vein undetectable , No thyroid enlargement
- Chest
  - Inspection:
    - Normal and symmetric respiratory movement , No deformity , No operation scar , No spider angiomata , Normal breast
    - No gynecomastia
  - Palpation:
    - No tender point , No palpable mass , No vocal fremitus
  - Percussion:
    - Normal dullness , No hyper-resonance
  - Auscultation:
    - No rales , No rhonchi , No wheezing , Stridor No stridor , No peripheral brochial sound , No friction rub
- Heart
  - Inspection: No visible apical impulse
  - Palpation:
    - Not palpable PMI , No thrill , No heave
  - Auscultation:
    - Regular heart beats , No ectopic beats , No S3 , No S4 , No murmur , No gallop rhythm , No opening snap , No click
- Abdomen
  - Inspection:
    - No distension , No striae , No caput medusae , No operation scar , No hernia
  - Auscultation:
    - Normal bowel sound , No bruit , No metallic sound
  - Percussion:
    - No central tympanic , No hepatomegaly
  - Palpation:
    - No tenderness , No rebounding pain , No palpable mass , No splenomegaly
- Back and spine
  - No deformity , No flank knocking pain
- Extremities
  - No deformity , No petechiae , No ecchymosis , No livedo reticularis , No acrocyanosis , No clubbing finger , No palm erythema , No pitting edema , Normal joint
- Genito-anus
  - No mass , No prolapse of rectum , No genital ulcer , No hemorrhoid , No perianal abscess
- Neurological examination
  - Consciousness:
    - E 4 V 5 M 6 , No cognitive change
  - Cranial nerve:

-
        - Pupil size: 2.5/2.5 mm
    - Meninges:
    - Motor:
    - Sensory:
    - Cerebellum:
    - Stance/gait:

GU CONDITION

- External genitalia
    - Urethral orifice : normal
    - Penis : normal
    - Scrotum and its contents : normal

DRE

- Female
- Seminal vesicles : impalpable
- Rectal exam
    - Not performed
    - Normal anal tone

手術日期及方法

- 手術日期及方法20201015 Abdominoperineal resection for neogrowth of anorectum (APR) Laparoscopy

住院治療經過

- After admission, pre- op survey was done and no significant contraindication was noted. Abdominoperineal resection for neogrowth of anorectum was performed on 10/15 smoothly. After the surgery, the patient keep ERAS diet. However, the patient complained about right ankle pain and swelling, suspicion of gout attack. Thus we prescribed NSAID for pain control. She started try bland II diet since 10/19. As the patient recovered gradually, we removed CVP, abdominal stitches and penrose tube on 10/22. She was discharged under stable condition and OPD f/u was arranged.

合併症

一般檢查

- 累積報告 :

DATE	TIME	ALB	TP	BIL.T	BIL.D	ALKP	AST	ALT	LDH	CHOL	TG	HDL-C	LDL	UA	NH3	AMY
1091015	1419			0.30			15	8								
1091017	0901			0.54	0.19		15	7								

DATE	TIME	WBC	RBC	HGB	HCT	MCV	PLT	BLST	NEUT	BAND	SEG	LYM	MON	EOS	BAS
1091013	2046	9890	3.21	8.0			196		82.5			8.6	7.6	1.1	0.2
1091015	1404	11310	3.12	8.7			140		94.1			2.7	3.0	0.0	0.2
1091017	0901	9160	3.29	9.0	27.9	84.8	128		81.4			9.9	6.9	1.6	0.2
1091020	0855	8350	3.42	9.4			190		91.4			2.9	4.8	0.9	0.0

DATE	TIME	NA	K	CL	CA	BUN	CREAT	Mg	P	CRP	ESR	PCT	LIPASE	LACTATE
1091013	2048	143	4.6			26	2.08							
1091015	1419	146	3.7	117	6.9	18	1.57							
1091017	0901	142	3.0			7	1.16							
1091020	0918	145	3.0		6.1	6	1.01	1.3	1.7					

DATE	TIME	SP-GR	PH	PROT	GLUC	KETON	BILI	UROB	OB	NITR	LEU	RBC	WBC	EPCEL
1091017	1041	1.010	6.0	Negative	Negative	Negative	Negative	0.2	Negative	Negative	Negative	0-2	2-5	0-1

特殊檢查

病理報告

其它

出院時情況

- 3.改門診治療

出院指示

-

藥名	劑量	劑量單位	用法	頻次	數量	數量單位	現況	天數	單位
Mopride tab 5mg	1	TAB	PO	TID	21	TAB	TH	7	天
Spironolactone-25	1	TAB	PO	BID	6	TAB	TH	3	天

- Lactam tab 500mg | 1 | TAB | PO | QID | 28 | TAB | TH | 7 | 天

出院計劃

主治醫師 2062F 蔣鋒帆 / 住院醫師 9972C 陳智翔

正式摘要(電子簽章：000000201497334)

W52 / W52

doctorRank:B
